# Diminished mental- and physical function and lack of social support are associated with shorter survival in community dwelling older persons of Botswana

**DOI:** 10.1186/1471-2458-7-144

**Published:** 2007-07-05

**Authors:** Thomas Clausen, Adrian O Wilson, Robert M Molebatsi, Gerd Holmboe-Ottesen

**Affiliations:** 1 University of Oslo, Institute of Psychiatry, Oslo, Norway; 2 The African Foundation for Research and Interdisciplinary Training in Ageing (AFRITA), Harare, Zimbabwe; 3 University of Botswana, Department of Sociology, Gaborone, Botswana; 4 University of Oslo, Institute of General Practice and Community Medicine, Oslo, Norway

## Abstract

**Background:**

Mortality rates for older persons in Botswana have been unavailable and little is known of predictors of mortality in old age. This study may serve as a precursor for more detailed assessments.

The objective was to assess diminished function and lack of social support as indicators of short term risk of death.

**Methods:**

A national population based prospective survey was undertaken in Botswana; twelve rural areas and three urban centers were included.

372 community-dwelling persons aged sixty years and over, were included; 265 were followed-up. Sixteen subjects were deceased at follow-up.

Subjects were interviewed and clinically assessed at home. Measures of cognitive function, depression and physical function and sociodemographic information were collected. Subjects were followed-up at average 6.8 months after baseline.

**Results:**

Overall mortality rate was 10.9 per 100 person years. Age-adjusted odds ratios (OR) for death during follow-up were; 4.2 (CI 1.4–12.5) and 3.6 (CI 1.0–12.7) for those with diminished physical- and cognitive function, respectively.

Indicators of limited social support; household with only 1 or 2 persons and eating alone, yielded age adjusted ORs of 4.3 (CI 1.5–12.5) and 6.7 (CI 2.2–20), respectively, for death during follow-up.

**Conclusion:**

Older community dwelling persons with diminished cognitive- or physical function, solitary daily meals and living in a small household have a significantly increased risk of rapid deterioration and death.

Health policy should include measures to strengthen informal support and expand formal service provisions to older persons with poor function and limited social networks in order to prevent premature deaths.

## Background

The risk of death of older persons in differing populations is generally dependent on specific pre-existing conditions such as cognitive impairment, depression, loss of physical function, poor nutritional status, loneliness and limited social networks [1-6]. Physiological decline and diminished physical capacity invariably accompany ageing in all societies. Functional decline and social isolation increase vulnerability in old age, and the ensuing neuro-endocrine stress responses can precipitate a further decline in health [2]. Determinants of mortality in old age in Western societies are well documented [7-10]. In Africa the determinants of mortality in old age have not been investigated. It could be argued that mortality and its predictors in older Africans would differ little from what is already known in the West, but this would be to discard different disease patterns, and cultural and lifestyle influences. Non-communicable diseases of old age are frequently discarded in development of public health strategies of developing countries; yet non-communicable diseases, mental health problems included, are among the most important causes of sickness, disability and premature mortality in these settings [11,12].

Cognitive impairment has been found in 9 % of older Botswana subjects (aged 60 years and over): this increased with age. 12 % of older Batswana were also categorised as 'hazardous' drinkers whilst 7 % were depressed [13,14]. Mental disorders of old age such as dementia and depression contribute in turn to diminished physical function [15].

In the developed world population ageing and the loss of function in older citizens is addressed through the provision of targeted systems of health and social care. Social care systems may differ between nations, but require the financial support of Governments; usually to provide a mix of cash transfers and formal care services, such as pension schemes, meals, home based care and ultimately institutionalisation [16].

By contrast, the long held belief that the informal, but 'traditional' support from the extended family in African countries such as Botswana, has allowed a presumption that this can be depended upon for provision of care to older persons. Perhaps unsurprisingly formalised care is uncommon or often non-existent in Africa; indeed Botswana is one of only a handful out of 54 African countries with some form of old age pension programme [17]. Older persons in Botswana are entitled to the same level of public health care as any of its citizens, yet no old-age-specific health care facilities exist to meet this requirement. There are no personnel specialising in geriatric care in the country, and in 1999 there were 82 psychiatric nurses out of the total of 5000 registered nurses [18]. A decentralised general primary health care system has recently been created, with the goal of ensuring that a local clinic, run by nurses, is within 15 km (considered to be within walking distance) of all citizens, the aged included. Current socio-demographic trends and socio-medical pressures such as those caused by HIV/AIDS, urbanisation, and labour migration are contributing to the dissolution of the 'extended family' and thus contribute to the isolation and marginalisation of older Africans. At the same time, the burden older persons also carry for the provision of care to their dependants increases paradoxically [19]. Older Africans are compelled to care for their HIV infected children and eventually their orphaned grandchildren. Older persons' increasing contributions to civic stability and development in Sub-Saharan Africa cannot be ignored in a context of population ageing which will gain momentum in Africa over the next decades, accompanied inevitably by the increasing burden of non-communicable diseases and mental disorders of old age [20].

Informal care, social support, mental disorder and functional capacity require further investigation as possible predictors of premature mortality in older Africans [13,21,22].

This study assesses mortality rates and defines predictors of short survival in a national longitudinal sample of community dwellers aged 60 years and older in Botswana.

### Objectives

1) To determine crude age specific mortality rates among older persons 60 years and older in Botswana.

2) To investigate whether diminished mental and physical function were associated with mortality in older persons.

3) To investigate whether lack of social support was associated with mortality in this population.

## Methods

### Sampling

In 1998 a national representative household survey of 1085 respondents 60 years and over was conducted in Botswana, which at the time had a total population of approximately 1.6 million. The sample was drawn in accordance with the distribution of the most recent general population census in 1991, and aimed to represent 1 % of the target population of 100.000 in the selected age-groups [23]. A multi-stage cluster sampling technique was used. The sampling frame comprised all eligible older persons resident in each of the selected localities. The number of respondents required from each of the selected localities was calculated by proportionate distribution and randomly selected from the eligible group. All major tribal groups were represented according to their proportionate distribution in the total population.

In the field, a random selection of the required number of respondents from the locality was performed by starting from an arbitrary point in the community and thereafter including older persons eligible for the study until the required quota was met. Information regarding the number of households visited with potential respondents not available for participation in the survey is not available.

Twenty-three questionnaires were excluded during data analysis owing to poor data quality; thus 1062 respondents were included in the final household sample.

In a clinical component of the national survey (referred to as the 'baseline medical survey') specific information pertaining to health, and physical and mental function was collected by the research team (the first author and a nurse) using a random sub-sample of subjects stratified by age and gender. The subjects underwent a standardised clinical examination at home of 60 to 90 minutes duration. The medical baseline survey was planned to cover 50 % (n = 543) of the household sample, however, a response rate of 72 % yielded 393 subjects. Twenty-one questionnaires were later excluded owing to incomplete data; thus 372 respondents were included in the final analysis. More detailed descriptions of the data-collection methods for the household and baseline medical fieldworks have been described previously [13,24,25].

After completion of the household and baseline medical fieldwork in 1998, a revisit, planned to cover all the 372 included subjects, was performed in 1999 at an average of 6.8 months (range 4–9 months) after baseline. One hundred and seven subjects (29%) were not available at follow-up, but of these 78 were confirmed by relatives or neighbours to be travelling or at work etc. For the remaining 29 subjects no further information could be confirmed.

### Ethics

Informed consent was obtained from each subject before the baseline assessment and the project was approved by the Botswana Research and Ethics Committee.

### Assessments

Trained interviewers administered questionnaires in the subjects' homes during the household survey. The clinical examinations consisted of face-to-face interviews and standardised clinical examinations in the subjects' homes.

Information about deceased persons in the follow-up survey was confirmed verbally by primary relatives or by neighbours.

### Socio-demographic characteristics

The subjects' ages were obtained from the National Identity Card, or other official identity papers if available.

Information on marital status was dichotomised as single (never married, divorced or widowed) or married. Education was classified according to a 'yes' or 'no' response to the question, "Did you ever attended school?", irrespective of the number of years of schooling. Socioeconomic status (SES) was based on an additive index of eleven dichotomised indicators (characteristics of dwelling and possession of a number of household items) previously described [13,24]. The index was used to demonstrate relative differences in SES in this population, as no income data were collected [24].

### Measurements of physical and mental function

The chair rise test (described by Guralnik et al.) was performed to assess lower body strength. Subjects were required to rise from a seated to a standing position, with hands placed across the chest. They were instructed to stand and sit down, five times, as fast as possible, and the time taken was recorded in seconds [26]. Subjects who were either unable or did not complete the test within 30 seconds were classified as having diminished physical strength.

Self reported ability to walk a distance of 400 m without assistance was recorded.

The Montgomery and Aasberg Depression Rating Scale (MADRS) was used to assess depression. A score above 20 (moderate to severe depression) was applied as the cut-off for analysis [27].

An adaptation of Folstein's Mini Mental State Exam (MMSE) was used, adapted to suit the older of Botswana and the high level of illiteracy [28]. Items that required drawing of a geometrical figure, and writing or reading were omitted (leaving a maximum score of 26 versus the maximum score of 30 of the original MMSE). The range of scores was 0–26 and the cut-off point for cognitive impairment was set conservatively at below 16. (This adapted MMSE has not been validated and provides only an indication of the level of possible cognitive impairment in this population as thoroughly discussed previously [13,24]).

### Indicators of lack of social support

Respondents who reported that they "usually eat meals alone" were assumed to have diminished social support and were categorised accordingly in the analysis. Similarly those living alone or in households with only one other resident were categorised as having diminished social support compared to the norm of multigenerational larger households of an average of six members.

### Statistical analyses

Data were analysed using SPSS for Windows, Version 14.0. Variables are presented using descriptive statistics. Differences between categorical variables were tested with Pearson Chi-square tests.

Risk estimates for death during follow-up were calculated as crude odds ratios with 95% confidence intervals. A logistic regression model was used to present age-adjusted odds ratios and 95% confidence intervals for death during follow-up.

Mortality is presented as crude age group specific percentages and as standardised mortality rates per 100 person years (with CI calculated according to standard procedures for computing 95% confidence intervals for incidence rates) for the total sample.

## Results

A comparison of the results across the three datasets; Household, Baseline and Follow-up, (table 1) shows similar distributions of basic characteristics.

**Table 1:** General characteristics: Baseline population compared with "follow-up" population and whole "household" population. % *(n)*.

Sociodemographics	Sub-groups	*(n)*	*(1062)*	*(372)*	*(249)*
			National Household	Medical Baseline	Follow-up
Gender	Male		52	51	52
	Female		48	49	48
Age group (yrs)	60–69		50	45	43
	70–79		27	30	32
	80+		23	25	25
# Mean age (yrs)			72.2	73.2	73.5
Marital status	Married		51	54	48
	Single		49	46	52
SES group	High		*	25	22
	Medium		*	48	50
	Low		*	27	28
Area of residence	Urban		25	25	24
	Rural		75	75	76
Education	Attended school		47	45	47
	No schooling		53	55	53
No. of people in household	1		7	8	8
	2		10	12	12
	3 or more		83	80	80
# Mean no. of people in household			6.4	6.2	6.1

*- data missing for household sample, as it was derived from Medical Baseline questionnaire only.

# Mean scores

The median age was 71 years for both the baseline and follow-up population.

The median number of people in the household was 6 in both the baseline and follow-up populations. Living alone is not rare as 8% live unaccompanied and a total of 20 % live alone or with only one other person in the household.

The majority of subjects lived in rural areas and more than half had never attended school.

About 50 % were married or lived with a partner; whereas the other half were single, dominated by widowed persons (2/3 of single were widowed).

During follow-up 249 subjects were located alive and interviewed, and 16 subjects were confirmed to have died by family members or neighbours, totalling to 265 subjects.

Age distribution of crude probability of death among those with confirmed information at follow-up was 6 % in total; (3.6% (60–69 yrs); 5.9% (70–79 yrs); and 10.1% (80+ yrs). A more conservative estimate of crude probability of death (n = 16) during follow-up based on the total baseline sample (n = 372), assuming all deaths are registered, was 4.3% (2.4% (60–69 yrs); 4.5% (70–79 yrs) and 7.5% (80+ yrs).

Total observation time at follow-up was 1762 months (n = 265). Overall mortality of the follow-up sample combined is 10.9 per 100 person years.

Social support, household size, mental disorders, and reduced physical strength are all significant discriminators for survival in this population. Marital status, SES, area of residence and level of education do not discriminate significantly for survival in this study (table 3).

**Table 3:** Indicators of differential survival in follow-up sample. %.

		Follow-up Survivors	Follow-up Deceased	p-value
(n)		(249)	(16)	

Social support	Takes daily meals alone	11	47	< 0.001
Household size	1 person	8	31	< 0.001
	2 persons or less	20	50	0.003
Psychiatric disorder	Cognitive impairment	8	25	0.017
	Depression	6	19	0.050
Reduced physical strength	Failed chair raise × 5	23	56	0.002
Walk 400 m	Unable without help	11	31	0.047
Education	Never attended school	53	50	0.37
Marital status	Single	49	67	0.056
SES	Low	28	38	0.35
Area of residence	Urban	24	38	0.47

P-value based on Pearson Chi-square test.

Age adjusted risks for death during follow-up are, as a trend, lower, but still similar to the calculated crude odds ratios' and the confidence intervals are relatively wide due to the low numbers of deceased.

Cognitive impairment and physical functional decline and limited social support are associated significantly with increase in risk of death in the short term (follow-up period was on average 6.8 months). Depression shows a non-significant trend of increased risk of mortality.

## Discussion

At least 20 % of older persons in Botswana live in relative isolation. This renders false the notion that older Africans are always taken care of by their extended family. The combined effects of absence of "helping hands" in old age and increasing physical and mental frailty increase the risk of a premature death.

Community dwelling older persons in Botswana with cognitive impairment, reduced lower extremity strength, solitary eating and/or living in a household alone or with only one other person have a significantly increased short term risk of death. Attention to critical functional decline as a precursor of rapid death has also previously been reported in Zimbabwe, but no specific determinants for this association were described [29].

Estimated mortality at nearly 11 per 100 person years for the entire study population is more than twice the mortality found among age-peers in several European countries [10].

Older persons in Western societies who eat alone have been found to be at nutritional risk [30,31], and this is also the case with older persons in Botswana [25]. Eating patterns are indicative of social interaction (which can be seen as proxy indicator for support) and most elders share meals with others if possible [32]. Older persons in Botswana are left increasingly with no one to share their meals and hence "eating alone" may be seen as an indicator of social isolation with its attendant risks.

The shorter survival of older persons with cognitive impairment and physical disabilities in Botswana may be a function of lack of care at the stage of critical function decline which in turn may lead to rapid deterioration and death. Short survival as a result of function decline will ultimately lead to a lower prevalence of mental disorders or physical disability in old age in Africa. Thus differential survival may be one explanation and contributing factor explaining the variation in dementia prevalence in different regions of the world, with lower rates in Africa, [12,33,34]. Similarly, this could explain the relatively low level of physical disability and dependence found among older Africans [13,21]. Another possible explanation is that older persons in Africa today are "strong survivors" through tough selection mechanisms during their lifetime. These strong survivors may be less prone to chronic diseases of old age which might explain a lower prevalence in Africa of conditions such as dementia.

Other explanatory factors of different levels of chronic diseases are differences in lifestyle such as diet and level of physical activity across regions of the world.

It is likely that future generations of older Africans will experience less extreme selection as a result of improvement in general standards of living and health care; at least in countries such as Botswana.

A mortality rate of around 11 per 100 person years among older persons in Botswana is high compared with other older populations, although no comparative data from elsewhere in Africa exist.

How can it be that older Africans with documented lower prevalence of chronic age related diseases die at more than twice the rate of European age peers?

The explanation may be that when older Africans cross the dividing line between independence and dependency they are left with little or no public or informal support; and thus they literally die. There may be no specific disease causing this high mortality; rather the sum of mortality from any cause resulting from lack of care and support when functional abilities are below a critical level.

### Limitations

The short period available for follow-up meant that the registered number of deceased in the observation time was low. The sample size limited the number of covariates appropriate for multivariate analyses, and thus only age could be adjusted for. However, this does not imply that other socio-demographic, health variables or confounders are of less importance. Whilst these results should be viewed with caution they should ideally be the precursor of more detailed future investigation.

Subject ageing was dependent on the reliability and accuracy of the national identity card. There is no evidence that reliable birth registrations took place in Botswana longer than thirty years ago; thus reported age may have been subject to some extent of observer bias.

Attention has previously been drawn to the absence of a validated MMSE for use in an African context, in Zimbabwe and Botswana [13,35]. Recent research on dementia in Nigeria points to a cross culturally validated diagnostic dementia tool [36], but this instrument was not available to us at the stage of planning and implementing, and we therefore can only report on indications of cognitive impairment based on the adapted MMSE. However, as a tool for assessing relative differences according to chosen variables it is viewed to be adequate.

This study cannot infer firm causal relationships of mortality in this population. In other older populations cardiovascular diseases and cancers are among the most prominent causes of death [10]. Here the design and sample size allow only an illustration of some of the social precursors and circumstances associated with death in old age in Botswana.

It has previously been shown that the medical baseline sample is similar to and representative of the national household sample [24,25]. Based on the similarities in the follow-up and the two other samples presented in this study from Botswana, it may be inferred that the results of this follow-up sample are nationally representative.

### Implications

The biological patterns of morbidity and predictors of mortality of old age may not differ dramatically between African countries and other regions of the world. But the public responses to increasing levels of chronic diseases and mental disorders in old age in Africa need to be further developed and adapted to suit the cultural context. With planning and effort premature death in isolation, whether resulting from a mental or physical decline, is avoidable and preventable, even in the context of widespread resource shortages. African policy makers must evolve strategies to handle the prospect of increasing future numbers of older persons, as well as for their present seniors [37].

Training health staff and allied professions in non-communicable disease including mental disorder is urgently required in Botswana, in order to equip health care workers with skills to detect and monitor these diseases in the future. Additional support programs for informal carers could be one solution to reduce the number of older persons with reduced function, presently left with no caring hands.

The predictors of mortality described here could well be reflected throughout other Sub-Saharan African countries, and be used to inform policy and provision of care. However, more detailed investigation of these associations is required, not only in Botswana but elsewhere in Sub-Saharan Africa.

## Conclusion

Older community dwelling persons in Botswana with reduced cognitive or physical function, have a significantly increased risk of death. The 'traditional' extended family fails to cater for support and care for their older family members in a significant number of instances.

Policy makers should target and strengthen formal care mechanisms and encourage informal care towards older citizens, especially those with mental disorders, functional decline and limited social support, in order to reduce avoidable premature mortality.

The total burden of mental disorders and especially declining cognitive function in old age constitute a major portion, expected to increase in the decades to come, of a population's total health burden, yet these seem to receive disproportionately little attention from health planners and policymakers in the developing world and a disproportionately poor allocation of health care resources.

## Competing interests

The author(s) declare that they have no competing interests.

## Authors' contributions

All the authors have contributed significantly in the process leading to this paper.

TC; Took part in conceptualisation of research project, data collection, analyses, and drafted the paper.

AOW; Took part in conceptualisation of the paper, analyses and in manuscript preparations.

RMM; Took part in conceptualisation of research project, data collection and in manuscript preparations.

GHO; Took part in conceptualisation of research project, analyses and manuscript preparations.

All authors have read the final version of the paper and approve its content.

**Table 2:** Follow-up population and deceased by age-group.

	Follow up (n)	Deceased (n)	% dead at follow-up	Mortality per 100 p.y.	95 % CI
**Overall**					
	265	16	6.0	10.9	10.4 – 11.4
**Age-group**					
60–69	111	4	3.6	6.7	5.7 – 7.7
70–79	85	5	5.9	11.3	10.4 – 12.2
80+	69	7	10.1	19.8	19.1 – 20.5

p.y. = person years

**Table 4:** Crude and age-adjusted death risk; odds ratios (OR), for death during follow up, according to indicators of differential survival

	Risk of death during follow up			
	Crude		Age-adjusted *	
	OR	CI	OR	CI
Small household (1–2 persons)	4.4	1.5–12.6	4.3	1.5–12.5
Eating meals alone	6.8	2.3–20.0	6.7	2.2–20.0
Cognitive impairment	4.0	1.2–13.7	3.6	1.0–12.7
Depression	3.6	0.9–14.0	3.4	0.9–13.2
Reduced lower extremity strength	4.4	1.6–12.4	4.2	1.4–12.5
Unable to walk 400 m	3.6	1.2–11.1	3.2	1.0–10.5

* Age adjustment by Logistic regression models (Separate model for each condition).

## Funding

The project was funded by the Norwegian Council of Universities/Centre for International University Cooperation (NUFU). TCs' doctoral grant was funded through the Norwegian Research Council.

## Pre-publication history

The pre-publication history for this paper can be accessed here:


